# Patient education for alcohol cessation intervention at the time of acute fracture surgery: study protocol for a randomised clinical multi-centre trial on a gold standard programme (Scand-Ankle)

**DOI:** 10.1186/s12893-015-0035-z

**Published:** 2015-05-01

**Authors:** Hanne Tønnesen, Julie Weber Egholm, Kristian Oppedal, Jes Bruun Lauritzen, Bjørn Lindegård Madsen, Bolette Pedersen

**Affiliations:** WHO-CC, Clinical Health Promotion Centre, Bispebjerg/Frederiksberg Hospital, University of Copenhagen, Copenhagen, Denmark; Clinical Health Promotion Centre, Department of Health Sciences, Lund University, Skåne University Hospital, Malmö, Sweden; Orthopedic Department, Hospital of Southern Jutland, University of Southern Denmark, Aabenraa, Denmark; Alcohol and Drug Research Western Norway, Stavanger University Hospital, Stavanger, Norway; Department of Orthopaedic Surgery, Bispebjerg/Frederiksberg Hospital, University of Copenhagen, Copenhagen, Denmark; Department of Orthopaedic Surgery, Hvidovre Hospital, University of Copenhagen, Copenhagen, Denmark

**Keywords:** Acute fracture surgery, Hazardous drinking, Alcohol cessation intervention, Postoperative complications, Patient education, Cost-effectiveness

## Abstract

**Background:**

Patients with hazardous alcohol intake are overrepresented in emergency departments and surgical wards. These patients have an increased risk of postoperative complications with prolonged hospital stays and admissions to intensive care unit after surgery. In elective surgery, preoperative alcohol cessation interventions can reduce postoperative complications, but no studies have investigated the effect of alcohol cessation intervention at the time of acute fracture surgery. This protocol describes a randomised clinical trial that aims to evaluate the effect of a new gold standard programme for alcohol cessation intervention in the perioperative period regarding postoperative complications, alcohol intake and cost-effectiveness.

**Methods/Design:**

Patients with hazardous alcohol intake undergoing ankle fracture surgery will be recruited into the trial from multiple orthopaedic wards at university hospitals in Denmark, Sweden and Norway. Included patients will be randomly allocated to either standard care or the gold standard programme aimed at complete alcohol abstinence before, during and 6 weeks after surgery. It includes a structured patient education programme and weekly interventions meetings at the orthopaedic outpatient clinic. Furthermore, patients are provided with thiamine and B-vitamins, alcohol withdrawal prophylaxis and treatment, and disulfiram to support abstinence. Alcohol intake is biochemically validated (blood, urine and breath tests) at the weekly intervention meetings and follow-up visits. Follow-up assessments will be conducted 6 weeks and 3, 6, 9 and 12 months after surgery for all patients. The effect of the gold standard programme will be assessed comparing the outcome measures between the intervention and control group at each follow-up point.

**Discussion:**

The study will provide new knowledge about how to prevent alcohol-related postoperative complications at the time of acute fracture surgery. If effective, the results will be a benefit for the clinical course, patients and society alike.

**Trial registration:**

The protocol is registered in ClinicalTrials.gov (Id: NCT00986791).

## Background

Alcohol intake is an independent risk factor for postoperative complications after major and minor operations, elective and emergency procedures for men and women [1]. Patients with hazardous alcohol intake are therefore at increased risk of general infections, wound and pulmonary complications, prolonged hospital stay and admission to the intensive care unit after surgery compared to abstainers or non-hazardous drinkers [2].

The poor surgical outcome is seen in patients with hazardous alcohol intake – even without liver disease, pancreatitis or other alcohol-related diseases [3]*.* The responsible pathophysiological mechanisms include a variety of subclinical dys-functioning organ systems, such as sub-clinical cardiac dysfunction, prolonged bleeding time and en extreme endocrine stress-response to the surgical intervention per se [1,4,5].

The postoperative complication rate is about doubled at an intake as low as > 2 drinks per day [6,7]. However, most of these patients do not have diagnoses commonly associated with alcohol misuse [8]. As all forms of excessive drinking increases the risk of trauma and hospitalisation, hazardous drinkers are overrepresented in emergency departments (ED) and surgical wards [7,9].

Surgical patients as well as patient with trauma seem very motivated to change their lifestyle including drinking habits [10-12]*.* Reviews found that at least two in three of these patients accept alcohol screening and two in three accept participation in alcohol interventions [13]*.* This period has been described as a ‘window of opportunity’, even though it may be very short. Furthermore, one in three reduces their alcohol intake spontaneously, when admitted to surgical wards [14].

Only three alcohol intervention studies have evaluated the effect on postoperative complications in high quality designs. Two randomised clinical trials (RCT) evaluated 4 and 8 weeks of preoperative intensive alcohol cessation intervention for patients drinking 60 g of ethanol per day or more aimed at reduction of postoperative complications. The intervention programme in both RCTs included empowerment to support complete alcohol abstinence, information, weekly visits, and prophylaxis of withdrawal symptoms and relapse with benzodiazepine and disulfiram (DIS) [15]. The compliance was very high, and a recent meta-analysis showed a significant effect on risk reduction and alcohol intake [16]. In contrast, a controlled clinical trial in general elective surgery could not show an effect of brief intervention (BI) aiming at alcohol reduction on postoperative complications [17]. BI consisting of feedback, information and advice have shown low or no effect on alcohol intake over time in general hospital settings and general practice [18-21]. Thus, the effect of BI on alcohol intake seems too small to have an effect on postoperative complications.

A review of cost-effectiveness and cost-benefit studies on screening and BI supported the use of alcohol screening and BI, though the effect was only minor [22]. In trauma patients screening and BI for alcohol problems are cost-effective regarding number of re-trauma [23].

No previous studies have investigated the effect on complications and costs of intensive alcohol cessation intervention at the time of acute surgery. A comprehensive 6-weeks gold standard programme for alcohol cessation intervention (GSP-A) was translated from the previous preoperative alcohol cessation studies as well as studies using the gold standard programme (GSP) [24] for smoking cessation intervention in surgical settings [25]. The GSP-A is also based on evidence from systematic reviews, results from a cross-sectional study on risk factors in a hospital population and patient preferences regarding alcohol intervention in relation to surgery [13,16,26-30].

The aim of this study is to evaluate the effect of the GSP-A aimed at 6 weeks complete alcohol abstinence in the perioperative period for hazardous drinking patients undergoing ankle fracture surgery regarding postoperative complications, alcohol intake and cost-effectiveness up to 12 months after surgery.

## Methods/Design

### Study design and setting

A randomised clinical multi-centre study will be conducted including adult (minimum 18 years) patients undergoing ankle fracture surgery and drinking 21 or more drinks (one drink equals 12 g ethanol) per week for at least 3 months before admission. Patients are consecutively recruited from multiple orthopaedic wards at university hospitals. The centres initially participating in the study are Bispebjerg/Frederiksberg, Hvidovre and Southern Jutland from Denmark, Lund from Sweden as well as Haukeland and Stavager from Norway.

Included patients will be randomly allocated to either the intervention group or control group (standard care). Patients in the intervention group will receive the GSP-A aimed at alcohol abstinence at the time of ankle fracture surgery and 6 weeks after. It includes a structured patient education programme and weekly interventions meetings initially at the orthopaedic ward and continued after discharge at the outpatient clinic (5 in total). Patients in the control group will receive standard care for patients with hazardous alcohol intake according to the clinical guidelines in the wards.

**Figure 1:**
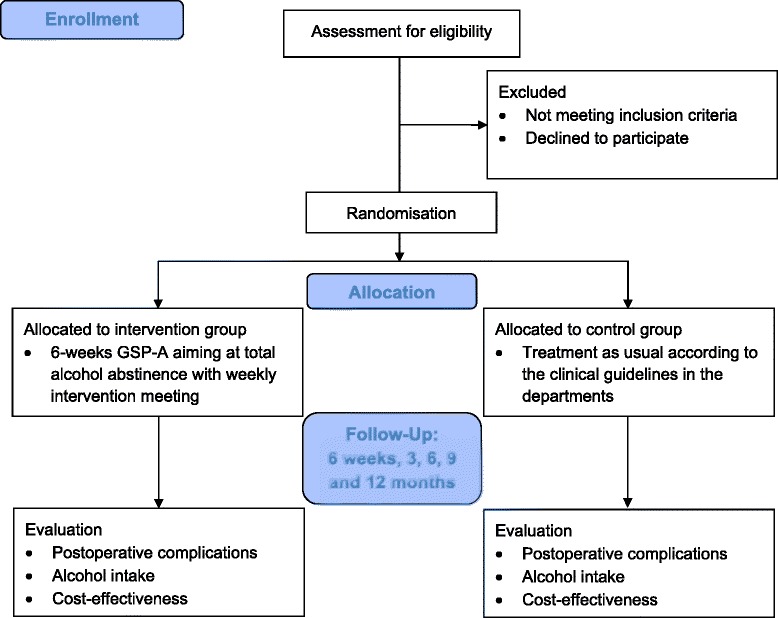
Trial profile.

For both groups follow-up assessments will be conducted after 6 weeks and 3, 6, 9 and 12 months after surgery; see trial profile in Figure 1. The primary outcomes are postoperative complications requiring treatment, alcohol intake (abstinence and non-hazardous drinking) as well as cost-effectiveness. The effect of the GSP-A will be assessed comparing the outcome measures between the intervention and control group at each follow-up point.

### Recruitment process and inclusion criteria

All ankle fracture patients admitted to the orthopaedic wards and scheduled for surgery are screened for eligibility by the research staff, and patients fulfilling the inclusion, but not the exclusion criteria, are invited to participate in the trial. The inclusion criteria are traumatic ankle fracture requiring internal fixation (osteosynthesis), informed consent, and randomisation within 36 hours after entering the orthopaedic ward. The fracture can be open or closed as well as simple or comminuted. The exclusion criteria are major trauma involving other fractures or major lesions, preoperative severe psychiatric disorders (including drug addiction, severe alcohol dependence [defined as experience of delirium or seizures during abstinence from alcohol] and dementia) or other conditions of reduced ability for giving informed consent; pathological fractures, pregnancy and lactation; allergy to benzodiazepines or DIS; uncompensated chronic diseases (including fulminant cardiac or liver insufficiency, which are contraindications for DIS) or ASA score 4-5 [31]; cancelled operation and withdrawal of informed consent.

Patients are included after informed consent, which can be obtained before or after the operation but within the 36 hours after admission. The patients can withdraw the informed consent at any time without any explanation and without consequences for their treatment or contact to the staff. For patients not giving or withdrawing their informed consent research staff will ask permission for follow-up via their medical recording solely for the purpose of the project.

### Randomisation, allocation concealment and sequence generation

A computer-generated list of random numbers will be used for allocation of the patients. The randomisation sequence is created using www.sealedenvelope.com statistical software, and is stratified by centre with a 1:1 allocation using random block sizes. The list is generated by a researcher not otherwise involved in the project and prior to the commencement of recruitment. The allocation sequence will be concealed from the research staff enrolling and assessing participants in sequentially numbered, opaque, sealed and stapled envelops that are impermeable to intense light. Corresponding envelops will be opened only after the patients have given informed consent and it is time to allocate the intervention group.

### Data collection

Baseline data will be collected for all included patients during admission in interviews: A detailed alcohol profile on intake over time (Timeline Follow-back [32]), ICD-10 criteria for alcohol dependency [33], test for alcohol use disorders (AUDIT-C [34]), alcohol withdrawal symptoms (CIWA-Ar [35]), breath test (ethanol during expiration), blood sampling for liver function (haemoglobin, alkaline phosphates, gamma-glyteryl-transferase, amino-transferases, bilirubine) and for alcohol markers (carbohydrate-deficient transferring, ethanol, phosphatidylethanol, mean cell volume, serum, EDTA–plasma and citrate-plasma); urine sampling for alcohol markers (ethyl glucuronide, ethyl sulphate, ethanol).

Furthermore, baseline characteristics regarding age and sex, socio-demographic factors, other lifestyle factors (smoking, overweight, risk of malnutrition and physical inactivity [36]), co-morbidity, self-rated ankle function, and self-evaluated health assessed in the SF-36 questionnaire [37] will be obtained for all patients.

During admission the following information will be collected from the medical records: AO-classification of the ankle fracture and surgical characteristics; ASA-score [31], operative procedure, antibiotics and thrombo-embolic prophylaxis, type of anaesthesia, blood loss, infusions, implants and surgical or anaesthetic problems. Besides, length of hospital stay, use of resources and medicines, discharge or transfer will be registered for all patients.

Follow-up visits will be conducted after 6 weeks, 3, 6, 9 and 12 months in the orthopaedic outpatient clinics. Patients will be evaluated on postoperative complications and second surgery, Olerud-Molander Ankle Score [38], dorsal plantar flexibility (after 3, 6, 9 and 12 months) and fracture status after 12 months confirmed by x-ray (satisfactory healing, secondary dislocation or non-union. Effect on alcohol intake will be assessed by Timeline Follow-back [32], alcohol withdrawal symptoms (CIWA-Ar [35]), breath test (ethanol during expiration) as well as blood sampling for liver function, alcohol markers and urine sampling for alcohol markers. Besides data on rehabilitation, readmissions, ED, general practitioners, specialist doctor, physiotherapist and occupational therapist, alcohol treatment services, nursing home, community nurse, home care, return to work and/or previous activity level, and use of medicines will be collected at all follow-up points.

All personal research data will be handled confidentially and anonymously after collection in the case report files. Only the study identification number and no personal identification data will be entered in the research database.

### Cost data

Data is collected for each individual patient in the project. The hospital costs will be based on the initial emergency room visits, the hospital stay in the orthopaedic surgical ward or intensive care units, readmissions and outpatient visits in the 12 months follow-up period. The costs related to the Scand-Ankle trial, including the project visits for outpatients, will be categorised as either GSP-A-related costs (staff salaries) or extra project costs (such as extra laboratory tests, medications and transportation). The costs related to primary care and other health care sectors will be derived from the consultation fees for general practitioners and “doctors on call”, average wages for specialists, home helpers, day care nurses, etc.

### Intervention

Patients allocated to the intervention group receive the GSP-A aiming at alcohol abstinence before, during and 6 weeks after surgery. The intervention will be initiated after the baseline interview has been completed and is conducted by research staff. To qualify for the intervention research personnel has to take part in a 2-day educational programme followed by practical training. The GSP-A consists of weekly intervention meetings at the orthopaedic ward or outpatient clinic (5 in total); see Table 1.

**Table 1:** **Gold standard programme for alcohol cessation intervention**

**Patient education programme**	
1.	First meeting (during admission): Level of motivation, ambivalence, pros and cons
2.	Second meeting (after 1 week): Dependence, withdrawal symptoms (experiences and expectations)
3.	Third meeting (after 2 weeks): Relapse (description and management)
4.	Fourth meeting (after 3 weeks): Benefits by short and long term alcohol abstinence
5.	Fifth meeting (after 4 weeks): Continued alcohol abstinence or reduced intake following intervention
**At each meeting**	
• Thiamine and B-vitamins (300 mg × 7 weekly)	
• Alcohol withdrawal prophylaxis and treatment (chlordiazepoxid 10 mg)	
• DIS support (200 mg × 2 weekly) supervised at weekly meetings (not administrated if patients test positive on an alcohol breath test)	
• Alcohol biomarkers (blood, urine and breath tests)	
• ECG	

The study medication is provided for free and transportation for the weekly meetings will be reimbursed. Patients can also contact the research personnel via phone or e-mail.

**Figure 2:**
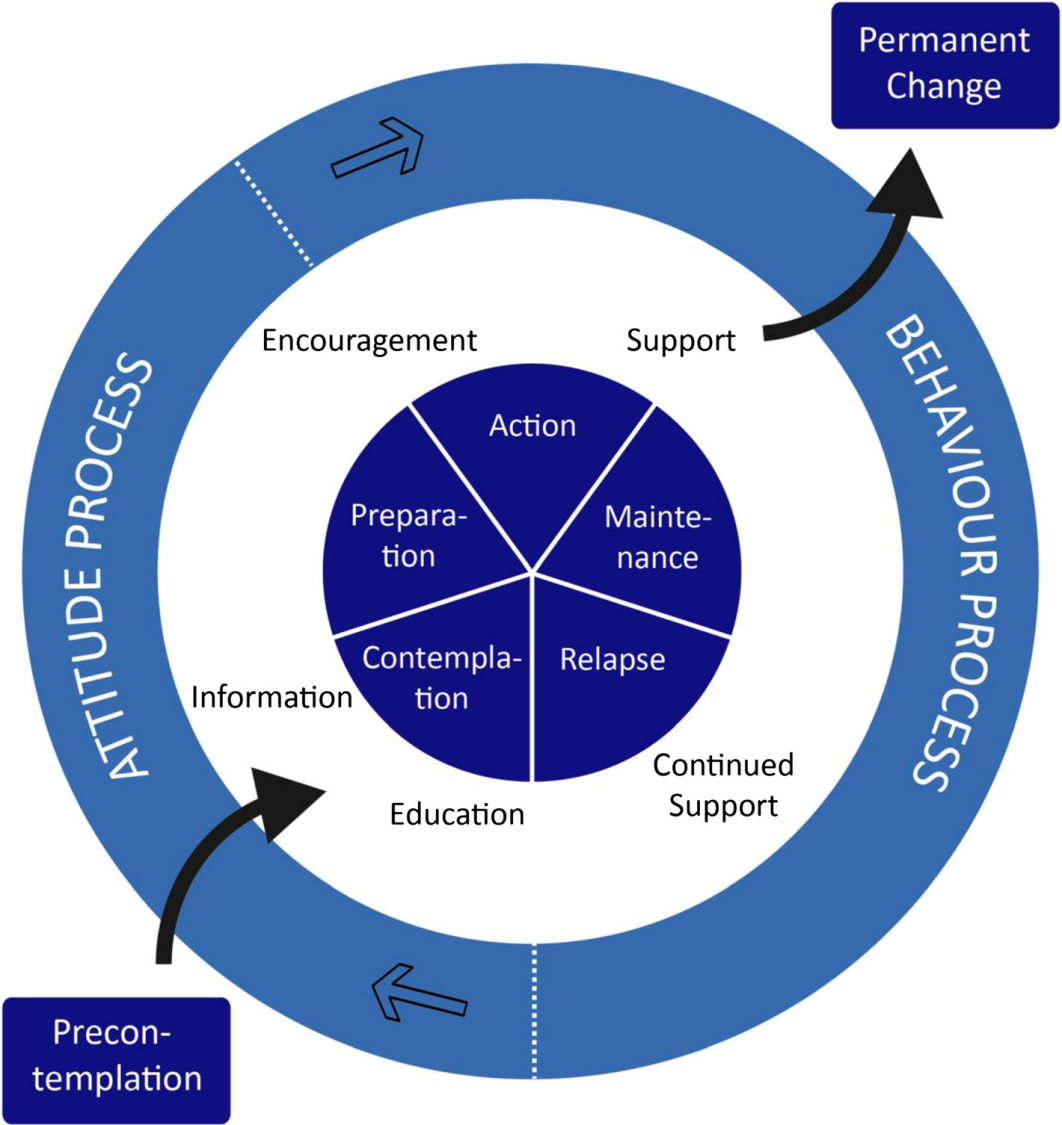
The stages of change model.

The structured patient education programme covers different topic at each meeting. At the first meeting, the patients will receive further information on the association between alcohol intake and postoperative complications. They will also be tested for ambivalence and level of motivation using different motivation scales to support patients’ empowerment: the LINE (see Table 2), the BOX (see Table 3) and the CIRCLE (see Figure 2) [39,40].

**Table 2:** **The LINE for identification**

**Information**	- All operations can cause complications, but many complications can be prevented. An important part of the prevention is your own effort as well as the support from the hospital.
	Question 1:
	- How important is it for you to prevent complications in relation to your surgery – on a scale from 0 to 10?
**Information**	- Hazardous drinking patients have 3 to 4 times more complications than others.
	Question 2:
	- How important is it for you to stop drinking alcohol in relation to your operation – on a scale from 0 to 10?

**Table 3:** **The BOX for assessingambivalence**

**What are the advantages of keep drinking alcohol?**	**What are the disadvantages of keep drinking alcohol?**
What are the disadvantages of giving up alcohol?	What are the advantages of giving up alcohol?

The education topic at the second meeting focuses on dependence and withdrawal symptoms with a particular focus on experiences and conceptions. The patients may develop stress caused by abstinence from alcohol. The withdrawal stress response is reduced by offering supportive medical treatment against development of mild to moderate withdrawal symptoms (minor doses of chlordiazepoxide), while severe alcohol withdrawal symptoms are treated in accordance with the recommendations and clinical guidelines from the psychiatric department related to the hospital. The personal stress is expected to be reduced by the empathic and empowering intervention. At the third meeting the focus is on relapse management and situations, where it is most likely that the patients will feel like drinking again. In case of relapse, the aim is to focus on the experiences the patients have gained while being abstinent and use these to initiate a new change and support the patients to resume abstinence. The patients are encouraged to participate in all meetings whether they comply with intervention or not. At the fourth meeting other benefits of short- and long-term alcohol abstinence is discussed with the patients, and at the last meeting the patients are encouraged to either continued alcohol abstinence after the 6-week intervention period (especially for patients who have experienced withdrawal symptoms), or if they do not want to be abstinent to keep their intake at a non-hazardous level. Following the intervention the patients can be referred to outpatient alcohol treatment facilities on their request.

### Control

Patients allocated to the control group receive standard care for patients with hazardous alcohol intake undergoing ankle fracture surgery including surveillance/scoring for alcohol withdrawal symptoms and related treatment according to the clinical guidelines in the wards. The standard procedures regarding alcohol are often non-interventional and characterised by short information and advices about changing alcohol habits. All control patients are informed about the possibility for alcohol intervention in their neighbourhood by delivery of a folder.

### All patients

Both groups receive routine procedures regarding general patient information, thromboembolic prophylaxis and antibiotics, anaesthesia, surgical intervention and other procedures according to the clinical guidelines for the operation at the involved wards. All patients have benzodiazepines for pre-medication. Sufficient thiamine is administered prior to eventual infusion of glucose.

In case of incidental findings all necessary steps would be taken for related information, diagnosing, intervention and follow-up. Severely dependent patients, including previous alcohol withdrawal symptoms, are not included in the study, but are referred to conventional treatment and observation according to national and international clinical guidelines.

### Measures

The primary and secondary outcomes will be evaluated at follow-up points after 6 weeks, 3, 6, 9 and 12 months.

#### Primary outcomes

- Postoperative minor and major complications requiring treatment: Wound complications, dislocated fracture, mal-union and secondary surgery and others such as pneumonia, thrombosis and neurological complications. Postoperative complications will be evaluated by an orthopaedic specialist blinded to patients’ group allocation.
- Continuous alcohol abstinence and non-hazardous drinking validated biochemically
- Cost-effectiveness

#### Secondary outcomes

The secondary outcomes include length of stay, reconvalescense (time until returning to work), self-evaluated health (SF36), degree of alcohol withdrawal symptoms and cost-effectiveness regarding changes in health-related quality of life (QALY [41]).

### Sample size

Sample size calculations are restricted by this being the first study evaluating the effect of alcohol cessation intervention in acute fracture surgery, and the number of patients needed differ depending on the outcome.

The effect on alcohol abstinence is based on the preoperative intensive alcohol interventions, which showed a very high effect above 90% in the intervention groups versus less than 10% in the control groups [16]. However, a more moderate effect is expected in acute fracture surgery with 50% abstainers in the GSP-A group after 6 weeks. This corresponds to a minimum of 2×12 patients.

Postoperative complication rates among ankle fracture patients were 30% for patients drinking at least 5 drinks per day versus 10% in patients without hazardous alcohol intake in a case-control study [42]. If similar complication rates after 6 weeks are expected in the control and GSP-A group respectively, a total maximum of 2×59 patients should be recruited over the 3 to 4 years inclusion period.

The number of patients for the cost-effectiveness analyses is based on the expected effect on the clinical outcomes [43].

All sample size calculations are based on a power of 80% and a risk of type 1 failure on 5%. Also to reduce differences between patient populations, each centre should include about 20 patients.

### Statistical analyses

The analyses will be performed blinded. The groups are compared regarding primary and secondary outcomes using Chi-square/Fisher’s exact test for frequencies and Mann-Whitney test for continuous outcomes. Analyses will be conducted using intention-to-treat principles. A p-value < 0.05 is considered statistically significant. Subgroup analyses for determination of prognostic factors are done through logistic regression analyses.

The cost-effectiveness of the GSP-A will be estimated by comparing the incremental cost to the incremental effect of the two groups. The GSP-A is considered more cost-effective than standard care if it is less costly and more effective. If the GSP-A is more costly and more effective, the additional cost per abstainer has to be considered worth paying [44].

Data will be analysed using IBM SPSS v. 19 and Excel 2010.

### Project status

Patient inclusion was finalised in 2014. A few patients are still in the follow-up process, and the analysis of short-term outcomes is still ongoing. The study is supposed to be completed by the end of 2015.

## Discussion

The overall aim of the Scand-Ankle project is to contribute to the development of evidence-based guidelines for perioperative alcohol cessation interventions, and this is the first study to evaluate the effect of a new comprehensive GSP-A on postoperative complications, alcohol intake and cost-effectiveness in acute fracture surgery.

A recent study shows that hazardous drinking patients with ankle fracture still have a very high complication rate (personal communications Aalykke et al.) similar to the rates used for the sample size calculation for this trial.

### Strengths and limitations

The randomised design provides evidence on the highest possible level regarding the effect of alcohol cessation intervention in acute fracture surgery. Blinding of patients and project staff is not possible or intended as the GSP-A includes patient education to support abstinence. However, evaluation of postoperative complications is performed blinded by an orthopaedic specialist unaware of the patients’ group allocation. Also, the statistical analyses are done blinded by an independent researcher.

Recruitment of patients to the trial is expected to be difficult, as the number needed to screen (NNS) to identify and include eligible patients may be very high. The NNS to get one eligible to accept participation in alcohol intervention studies varies from a few up to 70 patients [13]. The rates do not differ between RCTs and non-RCTs, or between brief and intensive alcohol intervention studies. Also, in a study on smoking cessation intervention in acute fracture surgery 61% of the eligible patients declined to participate [45]. Finally, recruitment rates may also vary between countries and/or centres.

### Perspectives

The study provides new knowledge about alcohol treatment and how to prevent alcohol-related postoperative complications as well as cost-effectiveness of these at the time of acute fracture surgery. From a clinical perspective this may help to improve the perioperative course. At the same time it will be necessary to address hazardous drinking among patients in acute fracture surgery, and thus also a need for systematic identification of alcohol intake, more detailed patient information as well as better education of staff. For the patients a shorter period of alcohol abstinence could be a positive experience and may also initiate a general reduction in hazardous drinking, which will be a benefit on long term. Also, the prospect of fewer complications is important from the patient perspective. For the society an optimised postoperative course, decreased use of health care resources and fewer sick days will induce immediate cost-savings, and in addition, fewer alcohol-related problems will also benefit society.

### Ethics approval and consent to participate

The Danish Scientific Ethical Committee System (CVK: 0908664), as well as the corresponding Ethical Committees in the Sweden (EPN Lund: 2009/9) and Norway (REK Vest: 2009/717), have all approved the project. The project has been approved by The Danish Data Protection Agency (2009-41-3741).

## Abbreviations

BI: Brief intervention
DIS: Disulfiram
ED: Emergency Department
NNS: Number needed to screen
GSP-A: Gold standard programme for alcohol cessation intervention
RCT: Randomised clinical trial
